# Relationship between anosmia and hospitalisation in patients with coronavirus disease 2019: an otolaryngological perspective

**DOI:** 10.1017/S0022215120001851

**Published:** 2020-08-25

**Authors:** H Avcı, B Karabulut, A Farasoglu, E Boldaz, M Evman

**Affiliations:** Department of Ear, Nose and Throat Diseases, Istanbul Kartal Dr Lutfi Kirdar Training and Research Hospital, University of Health Sciences, Turkey

**Keywords:** Anosmia, Coronavirus, Pandemics, Smell Disorders

## Abstract

**Objective:**

A study was carried out to evaluate the relationship between anosmia and hospital admission in coronavirus disease 2019 patients.

**Methods:**

The clinical data of 1534 patients with confirmed coronavirus disease 2019 virus were analysed. The study was conducted with medical records of 1197 patients (78 per cent). The basic characteristics of patients and symptoms related to otolaryngology practice were examined. The patients were divided into two groups according to their follow up: an out-patient group and an in-patient group.

**Results:**

The majority of patients presented with anosmia (44.2 per cent), dysgeusia (43.9 per cent) and fever (38.7 per cent). Anosmia was observed in 462 patients (47 per cent) in the out-patient group, and in only 67 patients (31.2 per cent) in the in-patient group. Younger age (odds ratio = 1.05, 95 per cent confidence interval = 1.03–1.06) and the presence of anosmia (odds ratio = 2.04, 95 per cent confidence interval = 1.39–3) were significantly related to out-patient treatment.

**Conclusion:**

Anosmia could be a symptom in the clinical presentation of the coronavirus disease 2019 infection.

## Introduction

The novel coronavirus disease 2019 (Covid-19) is a rapidly spreading contagious disease that affects mainly respiratory, gastrointestinal, cardiovascular and neurological systems.^1^ It was first described in December 2019 in Wuhan, China.^1^ The disease occurs as a result of a highly infectious virus called severe acute respiratory syndrome coronavirus 2 (SARS-CoV-2). The virus spreads rapidly among individuals through respiratory droplets during sneezing and coughing when social distancing is not practised.^2^ The World Health Organization (WHO) announced the disease to be a pandemic in March 2020.^3^ At the time of writing, a total of 638 146 cases and 30 039 deaths have been reported by WHO due to Covid-19 virus.^4^

The diagnosis of Covid-19 disease is based on clinical symptoms, positive polymerase chain reaction test results and the presence of ground-glass opacities on computed tomography, with varying specificity and sensitivity rates.^5^ The main presenting symptoms of the disease are fever, dry cough, dyspnoea, rhinorrhoea, nasal congestion, tonsil oedema, enlarged cervical lymph nodes, sore throat, fatigue, headache, myalgia, arthralgia and diarrhoea.^2,6^ Currently, there is cumulating evidence for a relationship between an atypical presentation of the disease and the presence of sudden-onset anosmia or hyposmia.^7^ Studies from South Korea, China and Italy reported that a significant number of patients affected by the Covid-19 virus presented with hyposmia or anosmia complaints.^2^ The sudden loss of smell and taste is important from an otolaryngological perspective because these symptoms may be related to underestimated and infectious Covid-19 carriers.^8^

In a recent multicentre European study by Lechien *et al*., 85.6 per cent of the patients presented with olfactory dysfunction, and 79.6 per cent had anosmia.^7^ Moein *et al*. found that 98 per cent of patients with the Covid-19 virus had smell loss.^9^ In addition, Yan *et al*. suggested that the hospital admission rate may be negatively associated with the presence of anosmia or hyposmia (26.9 per cent *vs* 66.7 per cent) and dysgeusia (23.1 per cent *vs* 62.7 per cent) symptoms, compared to those patients managed on an out-patient basis.^10^

Although the presence of anosmia and gustatory complaints in the search for atypical symptoms of Covid-19 disease is promising, the proportion of Covid-19 positive patients exhibiting olfactory disturbances is still unknown and may vary depending on ethnicity.

This study aimed to determine the relationship between atypical symptoms and hospitalisation in patients with Covid-19 virus, as the findings may be useful in otolaryngologists’ daily practice.

## Materials and methods

The clinical data of 1534 patients with the Covid-19 virus confirmed via real-time reverse transcription polymerase chain reaction, who were admitted to our hospital between 11 March and 21 April 2020, were analysed. Ethical approval was obtained from the institutional review board of the hospital's local ethics committee (approval code: 2020/514/175/2).

Patients were included in the study if they were aged over 18 years, with real-time reverse transcription polymerase chain reaction confirmed Covid-19 infection in respiratory specimens, and were able to answer the questionnaire. The following criteria were defined as study exclusions: patients with olfactory or gustatory dysfunctions before the epidemic, patients without laboratory confirmed Covid-19 infection at the time of diagnosis, and patients who were in the intensive care unit at the time of the study. After the exclusion of incomplete data, the study was conducted with the medical records of 1197 patients (78 per cent).

The essential characteristics of patients, such as age, gender, smoking habit and medical history (diabetes mellitus, hypertension, presence of chronic sinusitis, rhinitis, cardiac problems, chronic obstructive pulmonary disease (COPD) and asthma), were recorded. The presence of symptoms related to otolaryngology practice, including fever, cough, shortness of breath, sputum, nasal obstruction, rhinorrhoea, sore throat, post-nasal drip, anosmia and ear pain, was determined. Other symptoms likely to be related to the disease, such as dysphagia, fatigue, myalgia, abdominal pain, headache, vomiting, diarrhoea and gustatory symptoms, were also recorded.

The patients were divided into two groups according to their follow up: an out-patient group and an in-patient group.

For asymptomatic patients with Covid-19 virus positivity, and with a blood oxygen saturation level of more than 90 per cent, supportive care was provided and hydroxychloroquine administration was started (200 mg twice a day (2 doses), then 100 mg twice a day for 5 days). Patients with Covid-19 virus pneumonia and with an oxygen saturation level of more than 90 per cent received supportive care and hydroxychloroquine administration plus azithromycin treatment. The patients with uncontrolled co-morbidities, resistant fever, an oxygen saturation level of less than 90 per cent and severe diarrhoea were hospitalised, in accordance with national guidelines issued by the Republic of Turkey Ministry of Health.^11^

### Statistical analysis

Data analysis was performed with SPSS statistical software, version 20.0 (SPSS, Chicago, Illinois, USA). All continuous data are presented as means and standard deviations. The categorical data are presented as numbers and percentages. A one-sample Kolmogorov–Smirnov test was performed to analyse the distribution of the continuous variables; logarithmic transformations were used where appropriate. The student's *t*-test was used for the analysis of parametric variables, and the Mann–Whitney U test was employed for non-parametric variables. The chi-square test was used to compare categorical variables.

Multivariate regression analysis was performed to analyse the relationship between hospital admission and presenting symptoms. A primary regression model was generated using a stepwise procedure and included all potential interaction variables. This model was constructed from independent variables achieving *p* = 0.10 during bivariate analysis, and then the best-fit model was generated without interaction variables. Hospital admission was considered as a dependent variable. The independent variables were: age, anosmia, hypertension, cardiac problems, fever, cough, shortness of breath, fatigue, headache, vomiting and diarrhoea. For all calculations, a *p*-value of less than 0.05 was considered statistically significant.

## Results

The study population comprised 700 (58.5 per cent) male and 497 (41.5 per cent) female patients. The most common observed co-morbidity was hypertension, with a rate of 11.4 per cent (Table 1).

**Table 1.** Basic characteristics of study population

Parameter	Patients (*n* (%))
Gender	
– Male	700 (58.5)
– Female	497 (41.5)
Smoking	
– None	906 (75.7)
– 1–10 per day	188 (15.7)
– 10–20 per day	75 (6.3)
– >20 per day	28 (2.3)
Diabetes mellitus?	
– No	1091 (91.1)
– Yes	106 (8.9)
Hypertension?	
– No	1060 (88.6)
– Yes	137 (11.4)
Chronic sinusitis?	
– No	1182 (98.7)
– Yes	15 (1.3)
Rhinitis?	
– No	1184 (98.9)
– Yes	13 (1.1)
COPD?	
– No	1191 (99.5)
– Yes	6 (0.5)
Asthma?	
– No	1160 (96.9)
– Yes	37 (3.1)
Cardiac problems?	
– No	1165 (97.3)
– Yes	32 (2.7)

COPD = chronic obstructive pulmonary disease

There were 301 (25.1 per cent) asymptomatic patients, despite real-time reverse transcription polymerase chain reaction positivity findings. The majority of the patients presented with anosmia (44.2 per cent), dysgeusia (43.9 per cent), fever (38.7 per cent), cough (36.8 per cent), fatigue (23.2 per cent) and myalgia (21.5 per cent) (Table 2).

**Table 2.** Presenting clinical symptoms of study population

Clinical symptoms	Patients (*n* (%))
*Otology related symptoms*	
Fever?	
– No	734 (61.3)
– Yes	463 (38.7)
Cough?	
– No	756 (63.2)
– Yes	441 (36.8)
Shortness of breath?	
– No	1014 (84.7)
– Yes	183 (15.3)
Nasal obstruction?	
– No	1078 (90.1)
– Yes	119 (9.9)
Rhinorrhoea?	
– No	1160 (96.9)
– Yes	37 (3.1)
Sore throat?	
– No	1026 (85.7)
– Yes	171 (14.3)
Post-nasal drip?	
– No	1175 (98.2)
– Yes	22 (1.8)
Ear pain?	
– No	1193 (99.7)
– Yes	4 (0.3)
Sputum?	
– No	1179 (98.5)
– Yes	18 (1.5)
Anosmia?	
– No	668 (55.8)
– Yes	529 (44.2)
*Other symptoms*	
Fatigue?	
– No	919 (76.8)
– Yes	278 (23.2)
Dysphagia?	
– No	1187 (99.2)
– Yes	10 (0.8)
Headache?	
– No	1024 (85.5)
– Yes	173 (14.5)
Myalgia?	
– No	940 (78.5)
– Yes	257 (21.5)
Abdominal pain?	
– No	1166 (97.4)
– Yes	31 (2.6)
Vomiting?	
– No	1128 (94.2)
– Yes	69 (5.8)
Diarrhoea?	
– No	1106 (92.4)
– Yes	91 (7.6)
Dysgeusia?	
– No	671 (56.1)
– Yes	526 (43.9)

There were 982 patients in the out-patient group and 215 patients in the in-patient group. The mean age was significantly higher in the in-patient group compared to the out-patient group (49.07 ± 13.91 *vs* 37.34 ± 12.08, respectively; *p* < 0.001). Regarding the gender distribution, 124 patients (57.7 per cent) were male and the remaining 91 (42.3 per cent) were female in the in-patient group. There were 722 (73.5 per cent) non-smokers in the out-patient group, while a higher non-smoker rate was observed in the in-patient group (85.6 per cent; *n* = 184). Considering systemic concomitant diseases, 50 patients (23.3 per cent) had diabetes mellitus, 63 (29.3 per cent) had hypertension, and 19 (8.8 per cent) had cardiac problems in the in-patient group, which reflected significantly higher rates than the out-patient group (*p* < 0.001, *p* < 0.001 and *p* < 0.001, respectively). There were no significant differences between the study groups in terms of gender, or the presence of chronic sinusitis, COPD or asthma (Table 3).

**Table 3.** Comparison of basic characteristics and medical history for each study group

Parameter	Out-patient group values	In-patient group values	*p*-value
Age (mean ± SD; years)	37.34 ± 12.08	49.07 ± 13.91	<0.001*
Gender (*n* (%))			
– Male	576 (58.7)	124 (57.7)	0.79
– Female	406 (41.3)	91 (42.3)	
Smoking (*n* (%))			
– None	722 (73.5)	184 (85.6)	0.001*
– 1–10 per day	170 (17.3)	18 (8.4)	
– 10–20 per day	66 (6.7)	9 (4.2)	
– >20 per day	24 (2.4)	4 (1.9)	
Diabetes mellitus? (*n* (%))			
– No	926 (94.3)	165 (76.7)	<0.001*
– Yes	56 (5.7)	50 (23.3)	
Hypertension? (*n* (%))			
– No	908 (92.5)	152 (70.7)	<0.001*
– Yes	74 (7.5)	63 (29.3)	
Chronic sinusitis? (*n* (%))			
– No	972 (99)	210 (97.7)	0.165
– Yes	10 (1)	5 (2.3)	
Rhinitis? (*n* (%))			
– No	975 (99.3)	209 (97.2)	0.018*
– Yes	7 (0.7)	6 (2.8)	
COPD? (*n* (%))			
– No	979 (99.7)	212 (98.6)	0.07
– Yes	3 (0.3)	3 (1.4)	
Asthma? (*n* (%))			
– No	954 (97.1)	206 (95.8)	0.306
– Yes	28 (2.9)	9 (4.2)	
Cardiac problems? (*n* (%))			
– No	969 (98.7)	196 (91.2)	<0.001*
– Yes	13 (1.3)	19 (8.8)	

*Indicates significant difference (*p* < 0.05). SD = standard deviation; COPD = chronic obstructive pulmonary disease

With regard to otolaryngology related symptoms, the following presenting symptoms were most common in the in-patient group compared to the out-patient group: fever (58.1 per cent *vs* 34.4 per cent, *p* < 0.001), cough (59.1 per cent *vs* 32 per cent, *p* < 0.001), shortness of breath (35.3 per cent *vs* 10.9 per cent, *p* < 0.001) and sputum (5.1 per cent *vs* 0.7 per cent, *p* < 0.001). Anosmia was observed in 462 patients (47 per cent) in the out-patient group, and in only 67 (31.2 per cent) in the in-patient group. There were no significant differences between the study groups in terms of the presence of dysgeusia, nasal obstruction, rhinorrhea, sore throat, post-nasal drip or ear pain.

Other symptoms were also evaluated. Fatigue (37.7 per cent *vs* 20.1 per cent), dysphagia (2.3 per cent *vs* 0.5 per cent), headache (26.5 per cent *vs* 11.8 per cent), myalgia (27.4 per cent *vs* 20.2 per cent), abdominal pain (5.6 per cent *vs* 1.9 per cent), vomiting (16.7 per cent *vs* 3.4 per cent) and diarrhoea (17.2 per cent *vs* 5.5 per cent) were all higher in the in-patient group compared to the out-patient group (Table 4).

**Table 4.** Comparison of presenting clinical symptoms for each study group

Parameter	Out-patient group (*n* (%))	In-patient group (*n* (%))	*p*-value
*Otolaryngology related symptoms*			
Fever?			
– No	644 (65.6)	90 (41.9)	<0.001*
– Yes	338 (34.4)	125 (58.1)	
Cough?			
– No	668 (68)	88 (40.9)	<0.001*
– Yes	314 (32)	127 (59.1)	
Shortness of breath?			
– No	875 (89.1)	139 (64.7)	<0.001*
– Yes	107 (10.9)	76 (35.3)	
Nasal obstruction?			
– No	880 (89.6)	198 (92.1)	0.271
– Yes	102 (10.4)	17 (7.9)	
Rhinorrhoea?			
– No	951 (96.8)	209 (97.2)	0.779
– Yes	31 (3.2)	6 (2.8)	
Sore throat?			
– No	835 (85)	191 (88.8)	0.149
– Yes	147 (15)	24 (11.2)	
Post-nasal drip?			
– No	967 (98.5)	208 (96.7)	0.095
– Yes	15 (1.5)	7 (3.3)	
Ear pain?			
– No	978 (99.6)	215 (100)	1
– Yes	4 (0.4)	0 (0)	
Sputum?			
– No	975 (99.3)	204 (94.9)	<0.001*
– Yes	7 (0.7)	11 (5.1)	
Anosmia?			
– No	520 (53)	148 (68.8)	<0.001*
– Yes	462 (47)	67 (31.2)	
*Other symptoms*			
Fatigue?			
– No	785 (79.9)	134 (62.3)	<0.001*
– Yes	197 (20.1)	81 (37.7)	
Dysphagia?			
– No	977 (99.5)	210 (97.7)	0.021*
– Yes	5 (0.5)	5 (2.3)	
Headache?			
– No	866 (88.2)	158 (73.5)	<0.001*
– Yes	116 (11.8)	57 (26.5)	
Myalgia?			
– No	784 (79.8)	156 (72.6)	0.019*
– Yes	198 (20.2)	59 (27.4)	
Abdominal pain?			
– No	963 (98.1)	203 (94.4)	0.002*
– Yes	19 (1.9)	12 (5.6)	
Vomiting?			
– No	949 (96.6)	179 (83.3)	<0.001*
– Yes	33 (3.4)	36 (16.7)	
Diarrhoea?			
– No	928 (94.5)	178 (82.8)	<0.001*
– Yes	54 (5.5)	37 (17.2)	
Dysgeusia?			
– No	548 (55.8)	123 (57.2)	0.707
– Yes	434 (44.2)	92 (42.8)	

*Indicates significant difference (*p* < 0.05)

In the binary logistic regression analysis, only younger age (odds ratio = 1.05, 95 per cent confidence interval (CI) = 1.03–1.06) and the presence of anosmia (odds ratio = 2.04, 95 per cent CI = 1.39–3) were significantly related to out-patient treatment. However, the presence of hypertension, cardiac problems, fever, cough, shortness of breath, fatigue, headache, vomiting and diarrhoea were all significantly related to hospital admission (Table 5).

**Table 5.** Binary logistic regression analysis of presenting symptoms affecting in-patient management

Parameter	Β (SE)	*p*-value	OR	95% CI	
				Lower	Upper
Age	0.05 (0.008)	<0.001	1.05	1.03	1.06
Hypertension	−0.59 (0.25)	0.02	0.55	0.33	0.91
Cardiac problems	−1.19 (0.44)	0.007	0.30	0.12	0.72
Fever	−0.65 (0.18)	0.001	0.52	0.36	0.75
Cough	−0.57 (0.19)	0.003	0.56	0.38	0.82
Shortness of breath	−1.05 (0.22)	<0.001	0.34	0.22	0.54
Fatigue	−0.53 (0.21)	0.01	0.58	0.39	0.87
Headache	−0.61 (0.23)	0.01	0.54	0.34	0.86
Vomiting	−1.07 (0.33)	0.001	0.34	0.17	0.65
Diarrhoea	−0.69 (0.29)	0.01	0.49	0.27	0.89
Anosmia	0.71 (0.19)	<0.001	2.04	1.39	3

Constant (SE): 3.73 (1.61). SE = standard error; OR = odds ratio; CI = confidence interval

## Discussion

The Covid-19 virus caused a rapidly spreading pandemic and adversely affected the entire field of life. Otolaryngologists may be at a very high risk of Covid-19 infection as they deal with problems of the upper respiratory tract, which serves as the main reservoir of the virus.^2^ In our study, the presence of anosmia was one of the leading symptoms on admission and was significantly associated with an out-patient follow up.

The available data in the literature indicate that patients who present with influenza-like symptoms and anosmia are 6–10 times more likely to test positive for Covid-19 infection.^10,12^ Moein *et al*. observed self-reported smell loss in 35 per cent of patients with Covid-19 disease.^9^ In another multicentre study, Lechien *et al*. reported that olfactory and gustatory dysfunctions were both associated with mild-to-moderate Covid-19 disease.^7^ They also found that 79.6 per cent of patients had reported anosmia. However, Mao *et al*. reported less hypogeusia (5.6 per cent) and hyposmia (5.1 per cent) symptoms in patients with Covid-19 disease compared to the previous studies.^13^ In our study group, 529 patients (44.2 per cent) presented with anosmia. These varying results may be due to differences between studies in terms of: the diagnosis of anosmia, patient ethnicity and methods for diagnosing Covid-19 disease.

Regarding Covid-19 patients, it is important to determine the appropriate patient group to be hospitalised and establish whether any reliable test can be utilised to estimate hospital admission. A recent study by Yan *et al*. reported that patients presenting with loss of smell were 10 times less likely to have in-patient management of Covid-19 disease.^10^ These authors also stated that self-reported anosmia and hyposmia symptoms, reports which were reliable in 90 per cent of cases, were not associated with any other risk factors known to be related to the disease.^10^ It was suggested that smell loss could be an independent risk factor and may be utilised as a marker for the detection of patients with a mild presentation of Covid-19 infection.^10^ In our research, the regression analysis similarly revealed a lower hospitalisation rate in patients with anosmia. A possible explanation is that anosmia complaints are seen in the early stages of the disease. The lower rates of anosmia in the in-patient group may be a result of decreased awareness of olfactory dysfunction associated with the presence of severe symptoms such as respiratory distress. The reduced viral burden associated with treatment and the activated local host immune response could be consistent with the recovery of olfactory function.

The mechanisms leading to olfactory dysfunction in patients with Covid-19 infection are still unknown. The current study data demonstrated that Covid-19 infects olfactory epithelia and causes highly localised inflammation of the olfactory cleft. This process may in turn result in a conductive olfactory loss.^10^ However, the inflammatory process and obstruction were not the only aetiological factors to explain the olfactory dysfunction. It was thought that the Covid-19 virus might invade the central nervous system through the olfactory nerves, resulting in loss of smell function.^7,14^

Viral infections commonly present with non-specific symptoms such as malaise, fever and dry cough at the prodromal phase of the disease.^15,16^ In a recent study evaluating the presenting symptoms of Covid-19 patients, fever (98 per cent), cough (76 per cent), dyspnoea (55 per cent), and myalgia or fatigue (44 per cent) were the most common presenting symptoms.^16^ In addition to the common Covid-19 infection symptoms, a minority of the patients affected with the Covid-19 virus had intestinal symptoms such as diarrhoea.^16,17^ In our study, the majority of patients presented with fever, cough, fatigue and myalgia. Moreover, in the regression analysis, fever, cough, fatigue, shortness of breath, headache, vomiting, diarrhoea and dysgeusia were significantly related to in-patient follow up.

- The novel coronavirus disease 2019 (Covid-19) is a rapidly spreading contagious disease
- There is cumulating evidence for the relationship between atypical presentation of the disease and sudden-onset anosmia or hyposmia
- Most patients presented with anosmia (44.2 per cent), dysgeusia (43.9 per cent) and fever (38.7 per cent)
- Younger age and the presence of anosmia were significantly related to out-patient treatment
- Anosmia could be one symptom in the clinical presentation of Covid-19 infection
- In-patients may have fewer smell loss complaints because of decreased awareness of olfactory dysfunction associated with the presence of severe symptoms

The most common concomitant diseases in patients with the Covid-19 virus were reported to be hypertension (30 per cent), diabetes (19 per cent) and coronary heart disease (8 per cent).^16^ Similarly, another report showed that the most frequent co-morbidities in patients with Covid-19 disease were hypertension (27 per cent), diabetes (19 per cent) and cardiovascular disease (6 per cent), with almost identical rates.^18^ In our study, hypertension and cardiac disease were significantly related to in-patient management. The presence of these co-morbidities can disrupt the balance of the immune system and make patients more susceptible to viral diseases.

Some studies have reported a relationship between gender and anosmia in viral diseases.^19^ In a meta-analysis, males accounted for 60 per cent of Covid-19 patients. In addition, other studies reported that Middle East respiratory syndrome related coronavirus and severe acute respiratory syndrome related coronavirus infection rates were higher in males than females.^19,20^ Similarly, in our study, more males had a viral infection, in line with the literature, although the difference was not significant. Together, these findings suggest that the X chromosome and sex hormones in females may play an essential role in adaptive immunity against viral infections.

A strength of our study is the inclusion of a large group of patients diagnosed with the Covid-19 virus. However, the present study has several limitations. Firstly, the nasopharyngeal samples were examined via a real-time reverse transcription polymerase chain reaction test for the detection of the Covid-19 virus. Although a real-time reverse transcription polymerase chain reaction test confirms the diagnosis of Covid-19 in the vast majority of cases, false positive or negative results are possible.^21^ To date, no completely reliable tests have been confirmed to detect the disease. Secondly, our study lacks reliable follow-up data. Future studies could evaluate the relationship between recovery time and treatment response in patients with olfactory dysfunctions.

## Conclusion

Our study demonstrated that anosmia could be one symptom in the clinical presentation of the Covid-19 infection. Based on our results, patients with sudden smell loss may be considered in the early and infectious stage of the disease, which is an important consideration for otolaryngologists.

## References

[1] Zhong BL, Luo W, Li HM, Zhang QQ, Liu XG, Li WT Knowledge, attitudes, and practices towards COVID-19 among Chinese residents during the rapid rise period of the COVID-19 outbreak: a quick online cross-sectional survey. Int J Biol Sci 2020;15:1745–52 10.7150/ijbs.45221 PMC7098034 32226294

[2] Krajewska J, Krajewski W, Zub K, Zatoński T. COVID-19 in otolaryngologist practice: a review of current knowledge. Eur Arch Otorhinolaryngol 2020;277:1885–97 32306118 10.1007/s00405-020-05968-y PMC7166003

[3] Ludwig S, Zarbock A. Coronaviruses and SARS-CoV-2: a brief overview. Anesth Analg 2020;131:93–6 32243297 10.1213/ANE.0000000000004845 PMC7173023

[4] Gov.UK. Guidance: COVID-19: infection prevention and control (IPC). In: https://www.gov.uk/government/publications/wuhan-novel-coronavirus-infection-prevention-and-control [27 March 2020]

[5] Li M, Lei P, Zeng B, Li Z, Yu P, Fan B Coronavirus disease (COVID-19): spectrum of CT findings and temporal progression of the disease. Acad Radiol 2020;27:603–8 32204987 10.1016/j.acra.2020.03.003 PMC7156150

[6] Wan S, Xiang Y, Fang W, Zheng Y, Li B, Hu Y Clinical features and treatment of COVID-19 patients in northeast Chongqing. J Med Virol 2020;92:797–806 32198776 10.1002/jmv.25783 PMC7228368

[7] Lechien JR, Chiesa-Estomba CM, De Siati DR, Horoi M, Le Bon SD, Rodriguez A Olfactory and gustatory dysfunctions as a clinical presentation of mild-to-moderate forms of the coronavirus disease (COVID-19): a multicenter European study. Eur Arch Otorhinolaryngol 2020;277:2251–61 32253535 10.1007/s00405-020-05965-1 PMC7134551

[8] European Rhinologic Society. In: https://www.europeanrhinologicsociety.org/ [27 March 2020]

[9] Moein ST, Hashemian SMR, Mansourafshar B, Khorram-Tousi A, Tabarsi P, Doty RL. Smell dysfunction: a biomarker for COVID-19. Int Forum Allergy Rhinol 2020;10:944–50 32301284 10.1002/alr.22587 PMC7262123

[10] Yan CH, Faraji F, Prajapati DP, Ostrander BT, DeConde AS. Self-reported olfactory loss associates with outpatient clinical course in Covid-19. Int Forum Allergy Rhinol 2020;10:821–31 32329222 10.1002/alr.22592 PMC7264572

[11] Republic of Turkey Ministry of Health. Current Status in Turkey. In: https://covid19.saglik.gov.tr/ [18 August 2020]

[12] Menni C, Valdes A, Freidin MB, Sudre CH, Nguyen LH, Drew DA Real-time tracking of self-reported symptoms to predict potential COVID-19. Nat Med 2020;26:1037–40 32393804 10.1038/s41591-020-0916-2 PMC7751267

[13] Mao L, Wang M, Chen S, Hu Y, Chen S, He Q Neurological manifestations of hospitalized patients with COVID-19 in Wuhan, China: a retrospective case series study. JAMA Neurol 2020;77:683–90 10.1001/jamaneurol.2020.1127 PMC7149362 32275288

[14] Koyuncu OO, Hogue IB, Enquist LW. Virus infections in the nervous system. Cell Host Microbe 2013;13:379–93 23601101 10.1016/j.chom.2013.03.010 PMC3647473

[15] Chan JF, Yuan S, Kok KH, To KK, Chu H, Yang J A familial cluster of pneumonia associated with the 2019 novel coronavirus indicating person-to-person transmission: a study of a family cluster. Lancet 2020;395:514–23 31986261 10.1016/S0140-6736(20)30154-9 PMC7159286

[16] Huang C, Wang Y, Li X, Ren L, Zhao J, Hu Y Clinical features of patients infected with 2019 novel coronavirus in Wuhan, China. Lancet 2020;395:497–506 31986264 10.1016/S0140-6736(20)30183-5 PMC7159299

[17] Habibzadeh P, Stoneman EK. The novel coronavirus: a bird's eye view. Int J Occup Environ Med 2020;11:65–71 32020915 10.15171/ijoem.2020.1921 PMC7205509

[18] Zheng YY, Ma YT, Zhang JY, Xie X. COVID-19 and the cardiovascular system. Nat Rev Cardiol 2020;17:259–60 32139904 10.1038/s41569-020-0360-5 PMC7095524

[19] Li LQ, Huang T, Wang YQ, Wang ZP, Liang Y, Huang TB COVID-19 patients' clinical characteristics, discharge rate, and fatality rate of meta-analysis. J Med Virol 2020;92:577–83 32162702 10.1002/jmv.25757 PMC7228329

[20] Channappanavar R, Fett C, Mack M, Ten Eyck PP, Meyerholz DK, Perlman S. Sex-based differences in susceptibility to SARS-CoV infection. J Immunol 2017;198:4046–53 28373583 10.4049/jimmunol.1601896 PMC5450662

[21] Singhal T. A review of coronavirus disease-2019 (COVID-19). Indian J Pediatr 2020;87:281–6 32166607 10.1007/s12098-020-03263-6 PMC7090728

